# No genetic causal association between dental caries and Alzheimer’s disease: a bidirectional two-sample Mendelian randomization analysis

**DOI:** 10.7717/peerj.15936

**Published:** 2023-08-23

**Authors:** Qiao Liao, Si-Zhuo Li, Fa-Fa Tian, Kun Huang, Fang-Fang Bi

**Affiliations:** 1 Department of Neurology, Xiangya Hospital, Central South University, Changsha, Hunan, China; 2 National Clinical Research Center for Geriatric Disorders, Xiangya Hospital, Central South University, Changsha, Hunan, China; 3 Department of Neurology, Fifth Affiliated Hospital of Sun Yat-Sen University, Zhuhai, Guangdong, China

**Keywords:** Dental caries, Alzheimer’s disease, Oral-microbiome-brain axis, Chronic inflammation, Bidirectional Mendelian randomization

## Abstract

**Background:**

An increasing number of observational studies have suggested an association between dental caries and Alzheimer’s disease (AD). The association between dental caries and Alzheimer’s disease may be mediated by confounders or reverse causality. In this study, we conducted bidirectional two-sample Mendelian randomization (MR) to estimate the bidirectional causality between dental caries and AD.

**Materials and Methods:**

Genome-wide association study (GWAS) summary statistics of dental caries were extracted from a published meta-analysis which included a total of 487,823 participants. GWAS datasets of AD and AD onset age were obtained from the FinnGen bank. A bidirectional two-sample analysis was performed to explore the causality between dental caries and AD.

**Results:**

For the dental caries-AD causality estimation, there was no significant association between dental caries and AD, neither with the AD GWASs from the FinnGen database (OR: 1.041, *p* = 0.874) nor with those from the International Genomics of Alzheimer’s Project (OR: 1.162, *p* = 0.409). In addition, the genetic susceptibility to dental caries was not related to the onset age of AD. No causality existed between dental caries and early-onset AD (OR: 0.515, *p* = 0.302) or late-onset AD (OR: 1.329, *p* = 0.347). For the AD-dental caries relationship, no causality was detected by the IVW method (OR: 1.000, *p* = 0.717). Findings from other MR methods were consistent. The pleiotropy test and sensitivity analysis confirmed the validity of these MR results.

**Conclusions:**

In this bidirectional MR study, robust evidence to support a bidirectional causal effect between dental caries and AD from the GWAS results within large-scale European-descent populations was absent. Having dental caries would not alter the onset age of AD.

## Introduction

Alzheimer’s disease (AD) is the main cause of dementia (Eratne et al., 2018). It is usually characterized by an insidious decline in cognitive function and gradual disease progression (Eratne et al., 2018). The deposition of amyloid plaques and the formation of neurofibrillary tangles are the cardinal features of AD. An epidemiological study of AD revealed that the prevalence is predicted to triple by 2050 as the population ages (Livingston et al., 2020; Scheltens et al., 2021). As there are still no curative therapies, AD still imposes huge emotional and economic burdens on individuals and society and remains a major threat to global health (Serrano-Pozo & Growdon, 2019). In recent years, significant advancements have been made in understanding the pathogenesis of AD, including the importance of neuroinflammation in AD, which cannot be ignored (Calsolaro & Edison, 2016).

Growing evidence suggests that oral microbiota disturbance could interrupt the immune defense of the host, leading to neuroinflammation by spreading from the oral cavity into the brain through the blood brain barrier (Kinane, Stathopoulou & Papapanou, 2017; Shoemark & Allen, 2015). Recently, disturbed oral microbiota profiles have been found to be related not only to systemic diseases but also to neurodegenerative diseases (Hajishengallis & Chavakis, 2021; Peng et al., 2022; Shen, 2020). The correlation between oral diseases and AD has been gradually noticed by many researchers (de Oliveira Araujo et al., 2021; Holmer et al., 2018; Orr et al., 2020). AD patients experience more prominent tooth loss and a higher burden of dental plaque (Wu et al., 2021). A case‒control study proved that AD patients have a higher incidence of dental caries and periodontal disease, more mucosal lesions, and worse saliva quantity and quality (Aragon et al., 2018). Other studies have shown that elderly people, especially those with dementia, are confronted with increased risks of developing high levels of coronal and root dental caries (Delwel et al., 2017; Ellefsen et al., 2009). Conversely, individuals exposed to dental amalgam fillings are faced with a higher chance of developing AD in a longitudinal study. A recent meta-analysis also confirmed that the presence of oral diseases is associated with a more rapid decline in cognitive function among AD patients aged 65 or older (Borsa et al., 2021), and a higher level of dental caries is related to more obvious cognitive deterioration (Ellefsen et al., 2008). However, some studies inconsistently hold the view that oral diseases are not related to the incidence of dementia (Holmer et al., 2022) or dementia in AD patients (Laugisch et al., 2021). Therefore, the relationship between oral diseases and AD remains to be clarified.

The causal relationship between oral diseases and AD could not be well explored by observational studies. Some disadvantages exist in these studies, such as limited sample size and a lack of randomization. To overcome these problems, Mendelian randomization (MR) has been proposed. MR is an analytic approach that uses genetic variants associated with exposure as instrumental variables (IVs) to estimate the causal effect of a modifiable risk factor (exposure) obtained from observational studies on an outcome (Burgess, Butterworth & Thompson, 2013; Liao et al., 2022). Genetic data usually come from large-scale genome-wide association studies (GWAS) since many large GWAS consortia have been built. Thus, MR has been a timesaving and cost-effective method to identify potential causal associations (Sekula et al., 2016). In the current study, bidirectional two-sample MR was performed to assess the bidirectional causality between oral diseases and AD in European populations.

## Materials and Methods

### GWAS summary statistics of dental caries

The summarized GWAS data of dental caries were obtained from a publicly acquired GWAS meta-analysis containing 487,823 participants (Shungin et al., 2019), which incorporated evidence from the Gene-Lifestyle Interactions in Dental Endpoints (GLIDE) consortium (Shungin et al., 2015) and UK Biobank (UKB) (Bycroft et al., 2018). All individuals were of European descent. The GLIDE consortium is a cohort-based project with detailed clinical measures to explore individual susceptibility or resistance to dental caries, while the UKB is characterized by self-reported measures of dental condition through questionnaires. In the GWAS meta-analysis, forty-seven single nucleotide polymorphisms (SNPs) with genome-wide significance were obtained for further analysis.

### GWAS summary statistics of Alzheimer’s disease

We only chose GWAS in the European population to remain in accordance with the population of dental caries. Therefore, genetic association estimates for AD were obtained from the FinnGen database (Kurki et al., 2022). The FinnGen consortium, launched in Finland, is a large public project that involves genome and health data from 500,000 Finnish biobank participants. We chose the following phenotypes from the FinnGen database: AD (AD, 3,899 cases and 214,893 controls), early-onset AD (ADEO, 587 cases and 214,885 controls) and late-onset AD (ADLO, 2,670 cases and 214,871 controls) (Kurki et al., 2022). The patients were divided into an early-onset group and a late-onset group according to whether they were older than 65 years based on the International Statistical Classification of Diseases (version 2016) (https://icd.who.int/browse10/2016/en). Furthermore, we also validated our MR results by using AD GWAS data from different databases, including data from the International Genomics of Alzheimer’s Project consortium (IGAP) and the UKB (Bycroft et al., 2018; Kunkle et al., 2019). The IGAP consortium, an authoritative consortium of AD, analyzed the genetic data of 63,926 European participants (21,982 AD cases and 41,944 controls) and reported more than 10 million SNPs. However, we finally decided to abandon the AD GWAS, which mostly harbored UKB participants (Larsson, Woolf & Gill, 2022), considering the influence of population overlaps between the exposure and the outcomes.

### MR design

**Figure 1:**
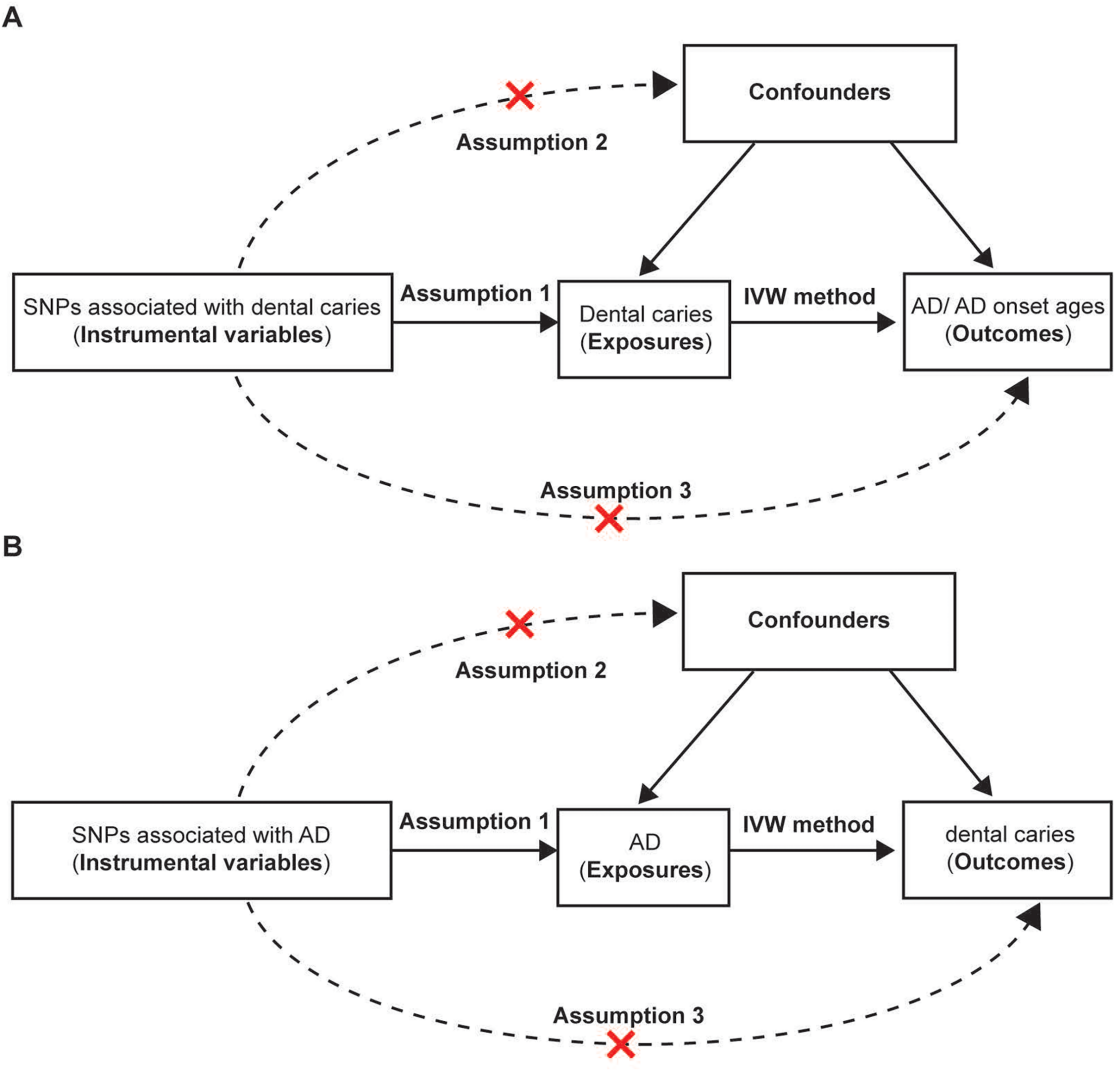
Assumptions and design of the bidirectional two-sample Mendelian randomization in our study. (A) The design of the mendelian randomization estimating the causality between dental caries and AD and AD onset ages. GWAS of AD and AD onset ages were ontained from the FinnGen bank. The causal effect between genetically predicted dental caries and AD were further confirmed by using AD GWAS from another authoritative datasets (International Genomics of Alzheimer’s Project). (B) The design of Mendelian randomization of causal effect of AD and dental caries. Three basic assumptions should be satisfied. Assumption 1, the chosen instrumental variables (IVs) should be strongly associated with the exposure; Assumption 2, no association should be found between the IVs and confounders; Assumption 3, the IVs could only influence the outcomes through the exposure rather than other pathways. SNPs, single nucleotide polymorphisms; AD, Alzheimer’s disease.

In our study, bidirectional two-sample MR was performed to investigate the causality between dental caries and AD (Fig. 1). Genetic variants were used as instrumental variables in the MR analysis. For the dental caries-AD association, SNPs associated with dental caries were used as IVs to explore the effect of dental caries on AD. SNPs associated with dental caries were obtained from a recent meta-analysis of large GWASs, and AD GWASs were obtained from FinnGen. Regarding the AD-dental caries association, SNPs associated with AD were used as IVs and were extracted from the GWAS from the IGAP consortium, while the dental caries GWAS was sourced from the UKB dataset. All the mentioned GWASs were conducted in European individuals.

### Selection of genetic instrumental variables

To determine whether the association between the risk factor (exposure) and the outcome is causal, the genetic variants associated with the modifiable factors were chosen as IVs in the MR, which are similar to the random assignment in a randomized trial (Emdin, Khera & Kathiresan, 2017). Additionally, the variants used as IVs should satisfy three basic requirements. First, the selected IVs are associated with the exposure (the relevance assumption). Second, there are no associations between the IVs and the confounders (the independence assumption). Third, the IVs could only exert an impact on the outcomes through exposure rather than other pathways (the exclusion restriction).

In the causal analysis of dental caries on AD risk, SNPs from GWASs that are significantly relevant with dental caries at the genome-wide level are used as IVs. To satisfy the first assumption, forty-seven SNPs are finally identified to be significantly associated with dental caries in a GWAS meta-analysis containing 487,823 participants and included in our MR analysis. Additionally, SNPs that were associated with factors that might influence the exposure (dental caries) or the outcome (AD) were removed to guarantee the second and third assumptions. Based on a literature review, smoking, obesity, hypertension and diabetes seem to be modifiable factors for AD (Durazzo, Mattsson & Weiner, 2014; Yu et al., 2020; Zhang et al., 2021). Therefore, these factors seem to be confounding factors that intervene in the causal relationship between dental caries and AD. Thus, we used the PhenoScanner database (http://www.phenoscanner.medschl.cam.ac.uk/) to manually screen and remove SNPs related to confounding factors and AD outcomes. Those SNPs that were directly associated with AD were manually removed as well. Consequently, nine SNPs (rs3865314, rs11676272, rs62106258, rs9366651, rs898797, rs10851907, rs8054556, rs28822480, rs7429279) related to those confounding factors were abandoned to minimize horizontal pleiotropy.

In the MR analysis of AD on dental caries risk, to satisfy the relevance assumption, we only included SNPs from large GWASs that were significantly associated with AD as IVs to investigate the causal relationship between AD and dental caries risk. To satisfy the independence assumption and the exclusion restriction, SNPs were dismissed if they were associated with confounders that might influence the risk of developing dental caries. Diabetes, sugar intake and education level were reported to be associated with the occurrence of dental caries (Martignon et al., 2021). Therefore, we manually removed SNPs that were associated with those factors based on the PhenoScanner database. Additionally, SNPs holding direct association with dental caries were also omitted.

We set genome-wide significance with a *p* value of 5 × 10^−8^ as a threshold to identify significant SNPs associated with dental caries used as IVs. A PLINK clumping method was used to prune SNPs in linkage disequilibrium (Purcell et al., 2007), and the clumping window of *r*^*2*^ was set as 0.001, while the distance was set as 10,000 kb. SNPs were sorted according to the *p* value, and the SNP with the lowest *p* value ranked first and was retained. Within a 10,000 kb distance, the SNP was dumped when its correlation with the most significant SNP was greater than 0.001. The genetic SNPs associated with the outcomes were also removed by searching the variant-trait associations on the public database PhenoScanner V2 (http://www.phenoscanner.medschl.cam.ac.uk/). In addition, the proxy would be taken as a substitute for SNPs that could not be found in the outcome GWAS according to LDlink (https://ldlink.nci.nih.gov/). To minimize the impact of confounding factors on the outcomes, we excluded SNPs associated with confounders based on PhenoScanner (Kamat et al., 2019). We extracted the remaining SNPs from the GWAS of outcomes with a minor allele frequency cutoff set as 0.01.

### Statistical analysis

Various MR analytic methods could be employed to estimate the causal effect using summarized statistics (Burgess, Butterworth & Thompson, 2013; Richmond & Davey Smith, 2022). Among them, the inverse variance weighted (IVW) method was the most commonly used method in MR analysis. Based on a meta-analysis approach, the IVW method was often used to obtain a pooled estimation of the ratio of the causal effect, especially when there were multiple IVs (Dudbridge, 2021; Richmond & Davey Smith, 2022). The MR‒Egger method was able to provide a test for a causal effect when the genetic variants were weak IVs, as well as to detect whether there was directional pleiotropy. When the MR‒Egger intercept value was not markedly different from zero, the MR‒Egger estimation would be in parallel with the IVW estimation (Burgess & Thompson, 2017). The median-based method was adopted as a supplement to the IVW method to reduce the bias produced by abnormal SNPs, and the maximum likelihood method was used if there was an overlap in the investigated population between the exposure and the outcome.

After MR analysis, heterogeneity and pleiotropy tests were performed in the sensitivity analysis. Cochran’s *Q* statistic was widely used to measure heterogeneity, and heterogeneity was present when the *p* value of *Q* was less than 0.05. A large *Q* value indicates that the SNPs associated with dental caries could exert various effects on outcomes even with large differences and that the findings should be cautiously interpreted. The MR‒Egger regression test was adopted to explore the directional pleiotropy of the dental caries-associated SNPs by comparing the intercept and the origin, and horizontal pleiotropy was absent if the *p* value was larger than 0.05. To detect any possible horizontal pleiotropy and identify any outliers, Mendelian randomization pleiotropy RESidual Sum and Outlier (MR-PRESSO) analysis was also employed (Verbanck et al., 2018). MR-PRESSO detected horizontal pleiotropy *via* the global test, identified pleiotropic variants *via* the outlier test, and then performed IVW to estimate the causal effect by removing the pleiotropic variants (Zhu, 2021). Leave-one-out analysis was also utilized to determine the potential SNPs that might impact dental caries-outcome associations.

We conducted our two-sample MR on R software (version 4.1.1) for Windows. Three packages were mainly employed for statistical analysis, data output and visualization: (1) ‘TwoSampleMR’ (https://github.com/MRCIEU/TwoSampleMR), (2) ‘MRPRESSO’ (https://github.com/rondolab/MR-PRESSO), and (3) ‘forestplot’ (https://cran.r-project.org/web/packages/forestplot/index.html). A *p* value was indicative of statistical significance based on a defined cutoff of 0.05.

## Results

### Instrumental variable selection

The SNPs that conformed with all three requirements were chosen as IVs to estimate the causal effect size between dental caries and AD. As demonstrated in Tables 1 and S1, for dental caries-AD association analysis, forty-seven genome-wide significant and independent SNPs that were associated with dental caries identified by the latest GWAS were selected. After the deletion of SNPs that did not satisfy the three consumptions and SNPs absent from the outcome GWASs from the FinnGen database, 30 SNPs remained in the analyses on AD, 30 SNPs in the analyses on ADEO and 30 SNPs on ADLO. Meanwhile, only 26 SNPs were incorporated into MR analyses based on the AD GWAS from the IGAP consortium.

**Table 1:** Bidirectional causal estimation between dental caries and Alzheimer’s disease.

Exposures	Outcomes	MR methods	Number of SNPs	*p* *	OR [95%C.I.]
Dental caries	AD (FinnGen)	IVW	30	0.874	1.041 [0.630–1.722]
		MR Egger	30	0.869	0.873 [0.177–4.298]
		Simple median	30	0.550	1.244 [0.607–2.549]
		Weighted median	30	0.986	0.993 [0.456–2.163]
	ADEO (FinnGen)	IVW	30	0.302	0.515 [0.146–1.817]
		MR Egger	30	0.901	1.292 [0.024–69.337]
		Simple median	30	0.151	0.276 [0.048–1.601]
		Weighted median	30	0.287	0.361 [0.055–2.354]
	ADLO (FinnGen)	IVW	30	0.347	1.329 [0.735–2.403]
		MR Egger	30	0.802	1.269 [0.200–8.061]
		Simple median	30	0.610	1.257 [0.521–3.034]
		Weighted median	30	0.775	1.137 [0.470–2.752]
	AD (IGAP)	IVW	26	0.409	1.162 [0.813–1.661]
		MR Egger	26	0.464	1.537 [0.495–4.775]
		Simple median	26	0.622	1.122 [0.710–1.772]
		Weighted median	26	0.979	1.006 [0.630–1.607]
AD	Dental caries	IVW	11	0.717	1.000 [0.999–1.001]
		MR Egger	11	0.859	1.000 [0.997–1.004]
		Simple median	11	0.723	1.000 [0.999–1.001]
		Weighted median	11	0.774	1.000 [0.999–1.001]

**Notes:**

* *p* value less than 0.05 is considered statistically significant.

MR, Mendelian randomization; SNPs, single nucleotide polymorphisms; AD, Alzheimer’s disease; ADEO, early onset Alzheimer’s disease; ADLO, late onset Alzheimer’s disease; FinnGen, GWAS data from the FinnGen database; IGAP, international genomics of Alzheimer’s project consortium; IVW, inverse variance weighted; OR, odds ratio; C.I., confidence interval.

For the AD-dental caries association analysis, after the process of clumping and filtering SNPs, 11 SNPs independently associated with AD taken from the IGAP consortium were ultimately retained for further analysis, and no SNP was retained from the FinnGen datasets. Thus, we only investigated the causal effect of AD on dental caries with GWAS data from the IGAP and UKB.

### Effect of dental caries on Alzheimer’s disease

**Figure 2:**
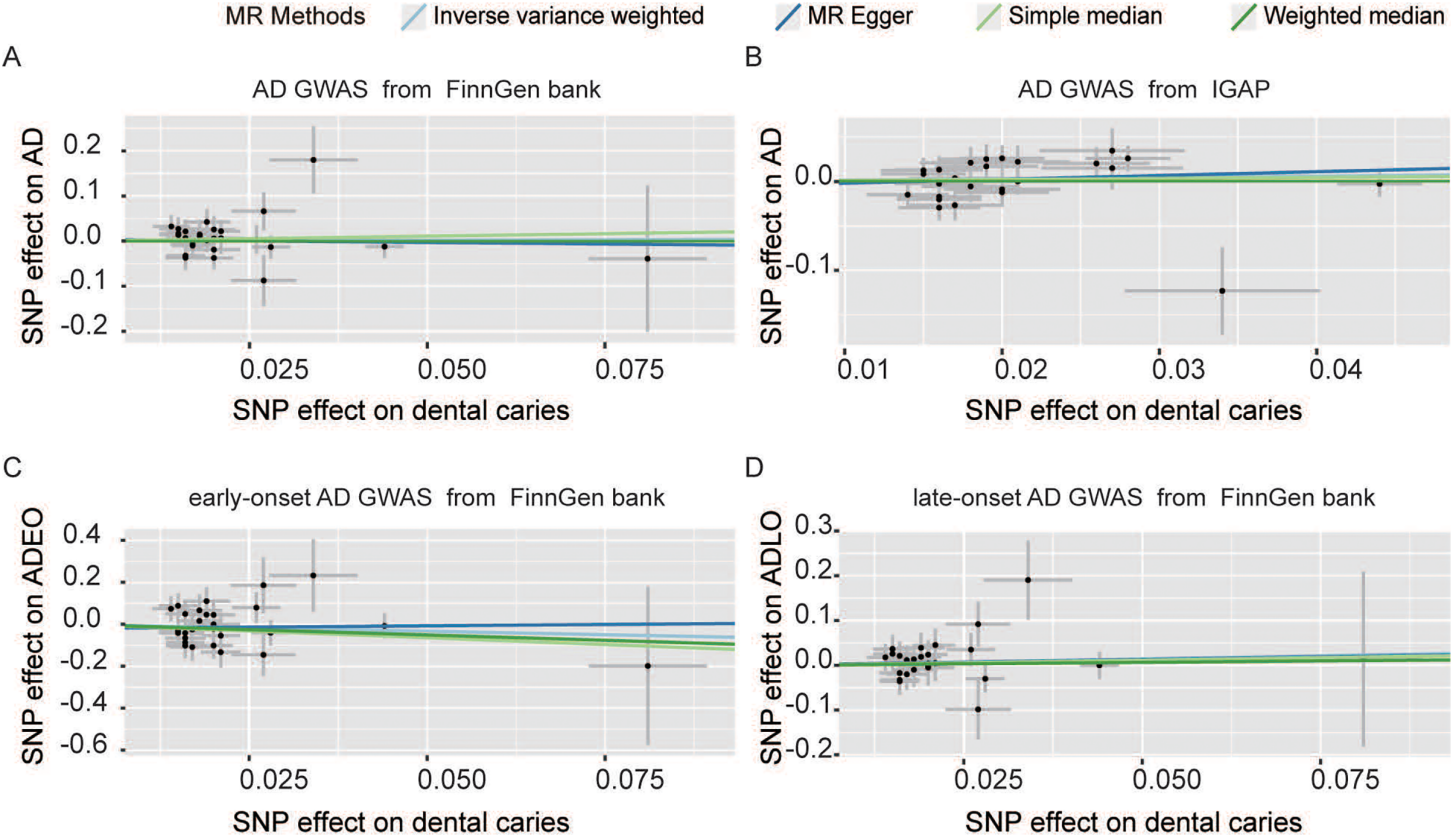
Scatter plot of causal effect of dental caries on AD (A and B) and AD onset ages (C and D). The outcome GWAS data of AD were sourced from FinnGen datasets (A) and IGAP (B) separately. SNPs, single nucleotide polymorphisms; AD, Alzheimer’s disease; ADEO, early onset Alzheimer’s disease; ADLO, late onset Alzheimer’s disease; FinnGen, GWAS data from the FinnGen database; IGAP, international genomics of Alzheimer’s project.

**Figure 3:**
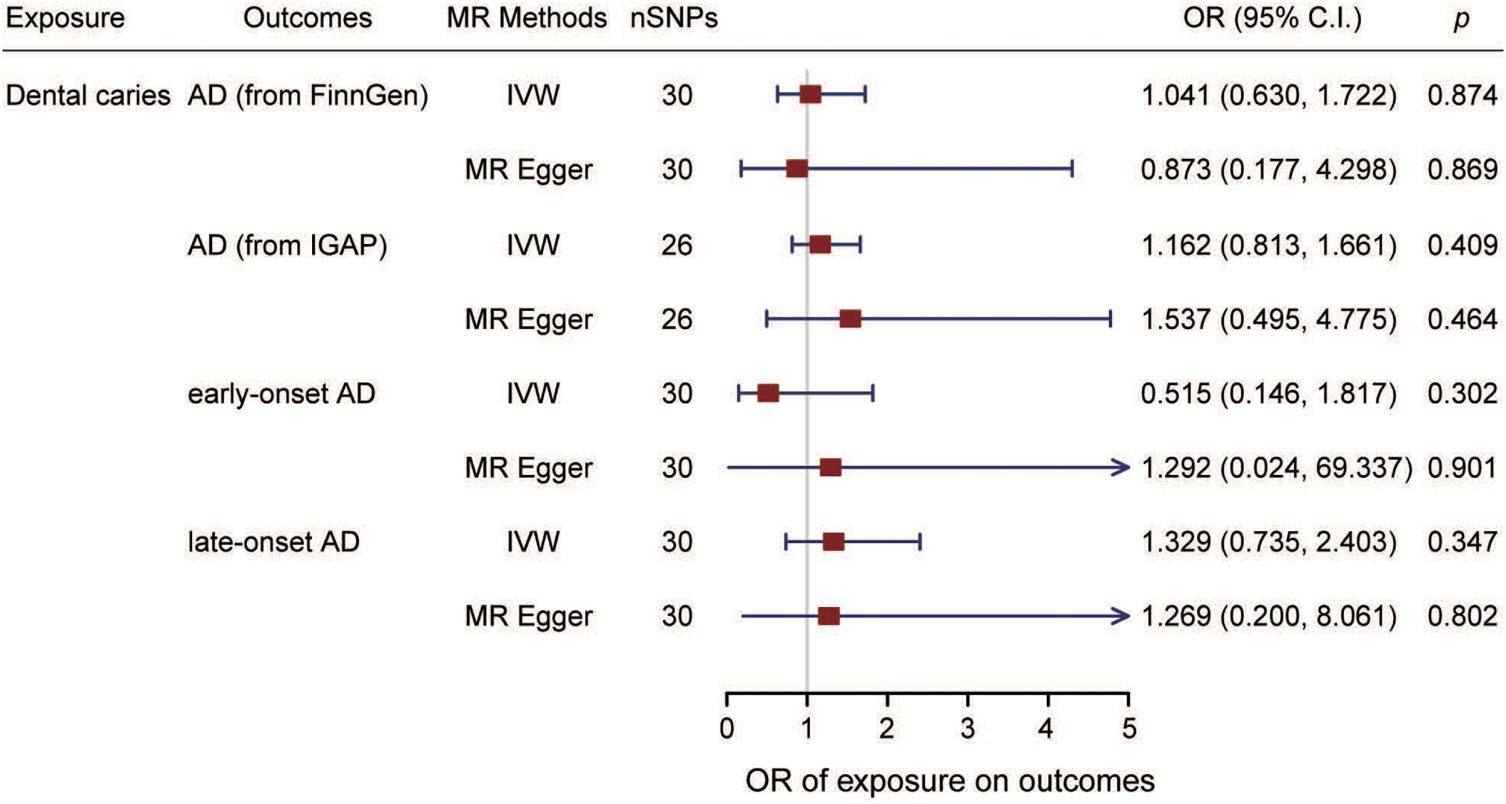
Forest plot of causality between dental caries and AD. The estimated causal effect of dental caries on AD and on the onset ages of AD. SNPs, single nucleotide polymorphisms; AD, Alzheimer’s disease; ADEO, early onset Alzheimer’s disease; ADLO, late onset Alzheimer’s disease; IVW, inverse variance weighted; OR, odds ratio; C.I., confidence interval.

The estimation of the effect of dental caries on AD according to the FinnGen from different MR methods is displayed in Fig. 2A. For the FinnGen dataset, as shown in Fig. 3, the causal estimation from the IVW method revealed that there was no significant association between dental caries and AD (odds ratio (OR): 1.041, 95% CI [0.630–1.722], *p* = 0.874). Moreover, as shown in Table 1, no obvious significance was identified from either the MR‒Egger or median method (MR–Egger OR: 0.873, 95% CI [0.177–4.298], *p* = 0.869; simple median OR: 1.244, 95% CI [0.607–2.549], *p* = 0.550; weighted median OR: 0.993, 95% CI [0.456–2.163], *p* = 0.986). In the sensitivity analysis, displayed in Table 2, there was no evidence of heterogeneity by Cochran’s *Q* statistic (*Q* = 29.530, *p* = 0.386). The MR‒Egger method also identified no significant direction pleiotropy by comparing the intercept value and zero (intercept value = 0.004, *p* = 0.821). Meanwhile, no robust horizontal pleiotropy was revealed by MR-PRESSO analysis (global test *p* = 0.467), and no outliers were detected.

**Table 2:** Sensitivity test of the bidirectional mendelian randomization analysis between dental caries and Alzheimer’s disease.

Exposures	Outcomes	MR methods	Heterogeneity test		Horizontal pleiotropy		MR-PRESSO
			Cochran’s Q	*p* *	Egger_intercept	*p* *	p* of global test
Dental caries	AD (FinnGen)				0.004	0.821	0.467
		MR-Egger	29.530	0.386			
		IVW	29.586	0.435			
	ADEO (FinnGen)				−0.020	0.637	0.255
		MR-Egger	33.551	0.216			
		IVW	33.825	0.246			
	ADLO (FinnGen)				0.001	0.959	0.738
		MR-Egger	24.280	0.667			
		IVW	24.283	0.715			
	AD (IGAP)				−0.006	0.614	0.149
		MR-Egger	34.226	0.081			
		IVW	34.598	0.096			
AD	Dental caries				2.24E-05	0.916	0.415
		MR-Egger	9.662	0.379			
		IVW	9.674	0.470			

**Notes:**

* *p* value less than 0.05 is considered statistically significant.

MR, Mendelian randomization; AD, Alzheimer’s disease; ADEO, early onset Alzheimer’s disease; ADLO, late onset Alzheimer’s disease; FinnGen, GWAS data from the FinnGen database; IGAP, International genomics of Alzheimer’s project consortium; IVW, inverse variance weighted.

MR analysis was also performed with the GWAS from the IGAP dataset to confirm the results. As shown in Figs. 2B and 3, no causal association between dental caries and AD risk was suggested (IVW OR: 1.162, 95% CI [0.813–1.661], *p* = 0.409). Consistent with the IVW results, evidence obtained from other MR methods also supported that the AD risk did not increase with increasing dental caries (MR‒Egger OR: 1.537, 95% CI [0.495–4.775], *p* = 0.464; simple median OR: 1.122, 95% CI [0.70510–1.772], *p* = 0.662; weighted median OR: 1.006, 95% CI [0.630–1.607], *p* = 0.979). No significant findings were identified from either Cochran’s *Q* heterogeneity test or the pleiotropy test with the MR‒Egger and MR–PRESSO methods (Table 2).

### Effect of dental caries on Alzheimer’s disease onset ages

The effect of dental caries on the onset age of AD was also investigated, and the scatter plot (Figs. 2C and 2D) demonstrated the relationship between dental caries and AD onset age. We found no causality between dental caries and the onset age of AD. Dental caries could not alter the onset age of AD. For the ADEO subtype, no apparent statistical evidence for the causal effect of dental caries was observed (IVW OR: 0.515, 95% CI [0.146–1.817], *p* = 0.302), as displayed in Fig. 3. In the meantime, the evidence for increased dental caries associated with a higher risk of ADLO was absent from the IVW approach (OR: 1.329, 95% CI [0.735–2.403], *p* = 0.347). Similar results were obtained by the MR‒Egger and weighted median methods either for the effect of dental caries on ADEO or on ADLO (Table 1). Next, we performed a sensitivity test to detect pleiotropy, and all the MR‒Egger intercepts were near zero (*p* = 0.637 for ADEO, *p* = 0.959 for ADLO), suggesting that there was no directional pleiotropy. The MR–PRESSO global test also detected no horizontal pleiotropy in ADEO and ADLO (*p* = 0.255 and 0.738, respectively).

### Effect of Alzheimer’s disease on dental caries

**Figure 4:**
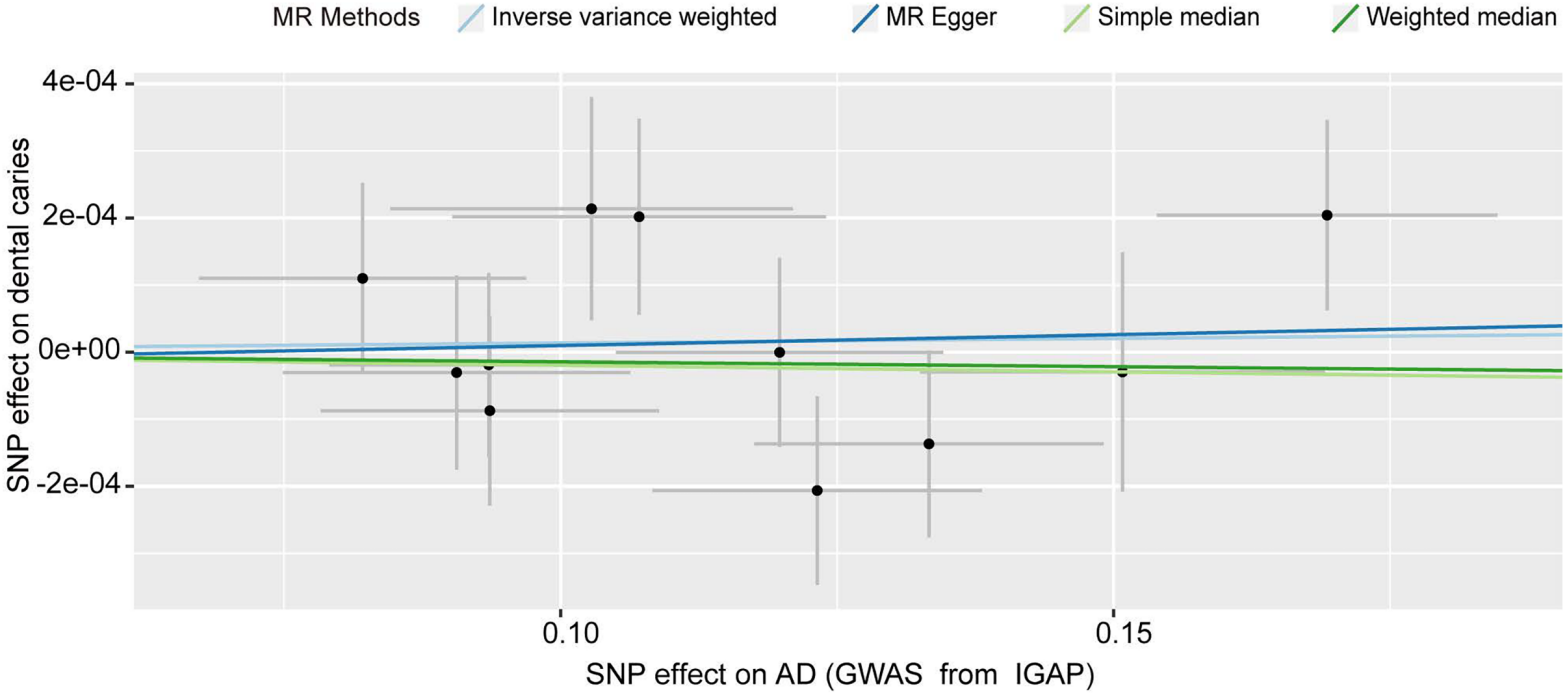
Scatter plot of the causal effect of AD on dental caries. The SNPs of AD and dental caries were extracted from summarized statistics from IGAP and UKB, respectively. SNPs, single nucleotide polymorphisms; AD, Alzheimer’s disease. IGAP, International Genomics of Alzheimer’s Project.

We detected no causal effect of AD on dental caries, and having AD did not alter the risk of developing dental caries (IVW OR: 1.000, 95% CI [0.999–1.001], *p* = 0.717) (Fig. 4). Consistent with the IVW results (Table 1), evidence obtained from other MR methods also supported that increased AD risk would not increase the chance of developing dental caries (MR‒Egger OR: 1.000, 95% CI [0.997–1.004], *p* = 0.859; simple median OR: 1.000, 95% CI [0.997–1.004], *p* = 0.723; weighted median OR: 1.000, 95% CI [0.999–1.001], *p* = 0.723). No significant findings were identified from either Cochran’s *Q* heterogeneity test (*p* = 0.379) or the pleiotropy test with the MR‒Egger (*p* = 0.916) and MR–PRESSO methods (*p* = 0.415).

## Discussion

To the best of our knowledge, this is the first study to explore the causal relationship between dental caries and AD by a bidirectional two-sample MR approach in individuals of European ancestry. Although there have been multiple observational studies implying an association between dental caries and AD, intriguingly, our results do not support bidirectional genetic susceptibility and causality between these two conditions in the European population. In simple terms, having dental caries will neither influence the risk of getting AD nor the onset age of AD. In addition, suffering from AD is also not the cause of dental caries.

In our MR analysis, to reduce pleiotropic bias against the results, we excluded SNPs directly related to the outcomes and SNPs that might impact outcomes *via* confounders. In the dental caries-AD association analysis, confounding factors were determined based on previous publications mentioning modifiable factors for AD. SNPs associated with smoking (Durazzo, Mattsson & Weiner, 2014), obesity, hypertension and diabetes were omitted to reduce pleiotropy (Livingston et al., 2020; Yu et al., 2020; Zhang et al., 2021). For the AD-dental caries association analysis, we eliminated SNPs related to risk factors for dental caries, including diabetes, sugar intake and education level (Martignon et al., 2021). In addition, advanced MR methods were employed to assess pleiotropic effects in our study. The weighted median estimator is an extension of the IVW and has a consistent estimation if only more than 50% valid IVs exist (Bowden et al., 2016). MR–PRESSO removes variants with potential pleiotropic effects, producing less biased estimation than IVW (Verbanck et al., 2018). Our MR sensitivity analysis showed that there were no significant pleiotropic effects, and our results were reliable.

There are several possible explanations for the inconsistent bidirectional causal associations reported in observational studies. One explanation for this discrepancy is that AD directly influences oral hygiene, thus resulting in dental caries (Aragon et al., 2018; Friedlander et al., 2006). With the gradual decline in cognitive function and motor function, the activities of daily living, including the ability to perform oral care in AD patients, decrease (Liao, He & Huang, 2022), which in turn increases the risk of dental caries and other oral diseases. On the other hand, the inflammatory mechanisms related to worse oral hygiene could exert an impact on AD (Singhrao et al., 2014; Wang et al., 2019). According to current knowledge of AD pathogenesis, inflammation is an important mechanism in disease onset and progression. Oral diseases are proposed to cause chronic inflammation and damage the brain by producing inflammatory cytokines which damage the intact blood‒brain barrier (Shoemark & Allen, 2015; Zhang et al., 2020).

A previous publication found that dental caries-related inflammatory burden is associated with a higher possibility of AD (Tiisanoja et al., 2019). Additionally, dental caries-related microbial dysbiosis, as well as the differential community of oral microflora between dental caries and AD, deserves to be noted. Differences in the flora might explain the absence of causality between dental caries and AD. It has been reported that patients with dental caries often have a relatively high abundance of *Streptococcus mutans* and *Lactobacillus fermentum* (Belstrom et al., 2017; Costalonga & Herzberg, 2014), and oral microbiome composition in AD patients is altered (Jungbauer et al., 2022; Wu et al., 2021). However, *Porphyromonas gingivalis* is more widely accepted as the keystone pathogen associated with AD. Bacteria and toxic proteases from *Porphyromonas gingivalis* have been found in the brains of AD patients (Dominy et al., 2019). Oral administration of *Porphyromonas gingivalis* in mice leads to the production of amyloid-β (a component of amyloid plaques) and tau pathology, reproducing the AD-related pathology in the brain (Dominy et al., 2019). Although our analysis did not support the causal effect of dental caries on AD, the role of the oral microbiota in the pathogenesis of AD cannot be ignored, and the importance of the oral-microbiome-brain axis in neurodegenerative disease should be noted (Narengaowa et al., 2021). We hypothesize that it is not the presence of oral diseases but the related microbial dysbiosis that is associated with AD; however, the specific mechanism is yet to be uncovered.

There are several major strengths of our MR study. In our bidirectional MR analysis, the GWAS data of dental caries were extracted from a meta-analysis of participants from the GLIDE consortium and UKB, and the GWAS data of AD came from the IGAP consortium and FinnGen datasets. Those data sources are trustworthy and authoritative, and we only included the robust SNPs that had genome-wide significance. In the current study, we established strict criteria to select IVs, and SNPs associated with confounders were abandoned to the utmost extent. In addition, various MR methods have been employed to reduce bias in causal estimation and strengthen causal inferences. No obvious heterogeneity, horizontal pleiotropy, or pleiotropic variants were identified by the sensitivity analysis, suggesting the validity and reliability of our results. Moreover, we validated our unidirectional MR findings by using an AD GWAS that included individuals from different genetic backgrounds who all had European ancestry as outcomes and arrived at similar conclusions.

Despite the validity and reliability of our MR results, there are several limitations in our study. First, since the individuals included in the primary studies of dental caries and AD GWASs were all from European populations, our findings may not be extended to other populations; therefore, our results should be interpreted with caution. Second, as our data were all derived from open databases, only summary statistics were available in our MR analysis, while the individual genetic data were inaccessible, which limited the detailed analysis of specific factors. Third, information about the proportion of familial and sporadic AD was absent, which might influence the causality estimation. Fourth, we only excluded SNPs associated with already known confounders such as smoking, diabetes and body mass index, and some other unknown confounders that might influence the dental caries-AD associations need to be considered. Finally, genetic IVs could only explain part of the exposure; the environment and lifestyles may also impact the exposure, and the mechanisms of how these genetic variants influence dental caries and AD were not explained by our study.

## Conclusion

In this bidirectional MR study, robust evidence to support a bidirectional causal effect between dental caries and AD from the GWAS results within large-scale European-descent populations was absent. Having dental caries would not alter the onset age of AD.

## Supplemental Information

10.7717/peerj.15936/supp-1 Supplemental Information 1 STROBE MR checklist.

10.7717/peerj.15936/supp-2 Supplemental Information 2 The IDs of GWASs and R codes.

10.7717/peerj.15936/supp-3 Supplemental Information 3 R scripts. The R scripts we used in our MR analysis on the causal relationship between dental caries and AD

10.7717/peerj.15936/supp-4 Supplemental Information 4 Genetic variants assocaited with dental caries.

## Abbreviations

AD: Alzheimer’s disease
MR: Mendelian randomization
IVs: instrumental varibale
SNP: single nucleotide polymorphism
GLIDE: gene- lifestyle interactions in dental endpoints
UKB: UK Biobank
FinnGen: GWAS data from the FinnGen database
IGAP: International Genomics of Alzheimer’s project
IVW: inverse variance weighted
OR: odds ratio
CI: confidence interval

## Additional Information and Declarations

### Data Availability

The following information was supplied regarding data availability: The genetic variants profiles used in the effect of dental caries on AD, IDs of GWASs in our study from the MRC IEU OpenGWAS project, and the R scripts are available in the Supplemental Files.
