# Supplementary material for: No genetic causal association between dental caries and Alzheimer’s disease: a bidirectional two-sample Mendelian randomization analysis
Source: PeerJ. 2023 Aug 23;11:e15936. doi: 10.7717/peerj.15936 (PMC10460150; doi:10.7717/peerj.15936)
Supplement: Supplemental Information 1 [file peerj-11-15936-s001.docx]

**STROBE-MR checklist of recommended items to address in reports of Mendelian randomization studies**^1^ ^2^

| **Item No.** | **Section** | **Checklist item** | **Page No.** | **Relevant text from manuscript** |
| --- | --- | --- | --- | --- |
| 1 | **TITLE and ABSTRACT** | Indicate Mendelian randomization (MR) as the study’s design in the title and/or the abstract if that is a main purpose of the study | 1,2 | Genome-wide association study (GWAS) summary statistics of dental caries were extracted from a published meta-analysis from a total of 487,823 participants. GWAS datasets of AD and AD onset ages were obtained from FinnGen bank. A bidirectional two-sample analysis was performed to explore the causality between dental caries and AD. |
|  | **INTRODUCTION** |  |  |  |
| 2 | **Background** | Explain the scientific background and rationale for the reported study. What is the exposure? Is a potential causal relationship between exposure and outcome plausible? Justify why MR is a helpful method to address the study question | 4-5 |  |
| 3 | **Objectives** | State specific objectives clearly, including pre-specified causal hypotheses (if any). State that MR is a method that, under specific assumptions, intends to estimate causal effects | 5 | In the current study, a bidirectional two-sample MR was performed to assess the bidirectional causality between oral diseases and AD in the European populations. |
|  | **METHODS** |  |  |  |
| 4 | **Study design and data sources** | Present key elements of the study design early in the article. Consider including a table listing sources of data for all phases of the study. For each data source contributing to the analysis, describe the following: |  |  |
|  | a) | Setting: Describe the study design and the underlying population, if possible. Describe the setting, locations, and relevant dates, including periods of recruitment, exposure, follow-up, and data collection, when available. | 6 |  |
|  | b) | Participants: Give the eligibility criteria, and the sources and methods of selection of participants. Report the sample size, and whether any power or sample size calculations were carried out prior to the main analysis | 6 |  |
|  | c) | Describe measurement, quality control and selection of genetic variants | 8 |  |
|  | d) | For each exposure, outcome, and other relevant variables, describe methods of assessment and diagnostic criteria for diseases | 6-7 | The patients were divided into early-onset group and late-onset group according to whether they were older than 65 years based on the International Statistical Classification of Diseases (version 2016) |
|  | e) | Provide details of ethics committee approval and participant informed consent, if relevant | 18 | Ethic statement were claimed in the involving GWAS studies. In details, consent of participants in the dental caries GWAS were obtained in primary studies. Written consent from participants in FinnGen were obtained on the Finnish Biobank Act. In the IGAP, written informed consent was obtained from study participants and the study protocols for all populations were approved by the Institutional review boards. |
| 5 | **Assumptions** | Explicitly state the three core IV assumptions for the main analysis (relevance, independence and exclusion restriction) as well assumptions for any additional or sensitivity analysis | 8 | Additionally, the variants used as IVs should satisfy three basic requirements. First, the selected IVs are associated with the exposure. Second, there are no any association between the IVs and the confounders. Third, the IVs could only exert impact on the outcomes through the exposure rather than other pathways. |
| 6 | **Statistical methods: main analysis** | Describe statistical methods and statistics used |  |  |
|  | a) | Describe how quantitative variables were handled in the analyses (i.e., scale, units, model) |  |  |
|  | b) | Describe how genetic variants were handled in the analyses and, if applicable, how their weights were selected | 8 |  |
|  | c) | Describe the MR estimator (e.g. two-stage least squares, Wald ratio) and related statistics. Detail the included covariates and, in case of two-sample MR, whether the same covariate set was used for adjustment in the two samples | 7,9 |  |
|  | d) | Explain how missing data were addressed | 8 | Besides, the proxy would be taken as a substitute for SNP that could not be found in the outcome GWAS according to the LDLink (https://ldlink.nci.nih.gov/) |
|  | e) | If applicable, indicate how multiple testing was addressed |  |  |
| 7 | **Assessment of assumptions** | Describe any methods or prior knowledge used to assess the assumptions or justify their validity | 8 |  |
| 8 | **Sensitivity analyses and additional analyses** | Describe any sensitivity analyses or additional analyses performed (e.g. comparison of effect estimates from different approaches, independent replication, bias analytic techniques, validation of instruments, simulations) | 9-10 | After MR analysis, heterogeneity and pleiotropy test were performed in the sensitivity analysis. |
| 9 | **Software and pre-registration** |  |  |  |
|  | a) | Name statistical software and package(s), including version and settings used | 10 | We conducted our two-sample MR on R software (version 4.1.1) for Windows. Three packages was mainly employed for statistical analysis, data output and visualization, listed as (1) ‘TwoSampleMR’ (https://github.com/MRCIEU/TwoSampleMR), (2) ‘MRPRESSO’ (https://github.com/rondolab/MR-PRESSO), and (3) ‘forestplot’ (https://cran.r-project.org/web/packages/forestplot/index.html). |
|  | b) | State whether the study protocol and details were pre-registered (as well as when and where) |  |  |
|  | **RESULTS** |  |  |  |
| 10 | **Descriptive data** |  |  |  |
|  | a) | Report the numbers of individuals at each stage of included studies and reasons for exclusion. Consider use of a flow diagram | Figure 1 |  |
|  | b) | Report summary statistics for phenotypic exposure(s), outcome(s), and other relevant variables (e.g. means, SDs, proportions) |  |  |
|  | c) | If the data sources include meta-analyses of previous studies, provide the assessments of heterogeneity across these studies |  |  |
|  | d) | For two-sample MR:  i.  Provide justification of the similarity of the genetic variant-exposure associations between the exposure and outcome samples  ii.  Provide information on the number of individuals who overlap between the exposure and outcome studies |  |  |
| 11 | **Main results** |  |  |  |
|  | a) | Report the associations between genetic variant and exposure, and between genetic variant and outcome, preferably on an interpretable scale | 14 | To the best of our knowledge, this is the first research to explore the causal relationship between dental caries and AD, by bidirectional two-sample MR approach in the European ancestry. Despite there has been multiple observational researches implying the association between dental caries and AD previously, intriguingly, our results do not support bidirectional genetic susceptibility and causality between these two conditions in the European population. |
|  | b) | Report MR estimates of the relationship between exposure and outcome, and the measures of uncertainty from the MR analysis, on an interpretable scale, such as odds ratio or relative risk per SD difference | 11-14 |  |
|  | c) | If relevant, consider translating estimates of relative risk into absolute risk for a meaningful time period |  |  |
|  | d) | Consider plots to visualize results (e.g. forest plot, scatterplot of associations between genetic variants and outcome versus between genetic variants and exposure) | Figure2,3,4 |  |
| 12 | **Assessment of assumptions** |  |  |  |
|  | a) | Report the assessment of the validity of the assumptions | 10,11 |  |
|  | b) | Report any additional statistics (e.g., assessments of heterogeneity across genetic variants, such as *I^2^*, Q statistic or E-value) | Table 2 |  |
| 13 | **Sensitivity analyses and additional analyses** |  |  |  |
|  | a) | Report any sensitivity analyses to assess the robustness of the main results to violations of the assumptions | 11-13, Table2 |  |
|  | b) | Report results from other sensitivity analyses or additional analyses | Table1 |  |
|  | c) | Report any assessment of direction of causal relationship (e.g., bidirectional MR) | 13 | We detected no causal effect of AD on dental caries, having AD would not alter the risk of getting dental caries (IVW OR: 1.000, 95% CI: 0.999-1.001, p=0.717) |
|  | d) | When relevant, report and compare with estimates from non-MR analyses |  |  |
|  | e) | Consider additional plots to visualize results (e.g., leave-one-out analyses) |  |  |
|  | **DISCUSSION** |  |  |  |
| 14 | **Key results** | Summarize key results with reference to study objectives | 14 | Despite there has been multiple observational researches implying the association between dental caries and AD previously, intriguingly, our results do not support bidirectional genetic susceptibility and causality between these two conditions in the European population. |
| 15 | **Limitations** | Discuss limitations of the study, taking into account the validity of the IV assumptions, other sources of potential bias, and imprecision. Discuss both direction and magnitude of any potential bias and any efforts to address them | 17 | Despite the validity and reliability of our MR results, there are several limitations in our study. |
| 16 | **Interpretation** |  |  |  |
|  | a) | Meaning: Give a cautious overall interpretation of results in the context of their limitations and in comparison with other studies | 14-15 |  |
|  | b) | Mechanism: Discuss underlying biological mechanisms that could drive a potential causal relationship between the investigated exposure and the outcome, and whether the gene-environment equivalence assumption is reasonable. Use causal language carefully, clarifying that IV estimates may provide causal effects only under certain assumptions | 15 |  |
|  | c) | Clinical relevance: Discuss whether the results have clinical or public policy relevance, and to what extent they inform effect sizes of possible interventions | 16 | Although our analysis did not support the causal effect of dental caries on AD, the role of oral microbiota could not be ignored in the pathogenesis of AD and the importance of oral-microbiome-brain axis in neurodegenerative disease should be noted. We hypothesize that it is not the presence of oral diseases but the related microbial dysbiosis that are associated with AD, however, the specific mechanism is await to be uncovered in the future. |
| 17 | **Generalizability** | Discuss the generalizability of the study results (a) to other populations, (b) across other exposure periods/timings, and (c) across other levels of exposure | 17 | since the individuals included in the primary studies of dental caries and AD GWAS were all European populations, our findings may not be extended to other populations, so our results should be interpretated with caution. |
|  | **OTHER INFORMATION** |  |  |  |
| 18 | **Funding** | Describe sources of funding and the role of funders in the present study and, if applicable, sources of funding for the databases and original study or studies on which the present study is based | 19 | This work was supported by the Science and Technology Innovation Program of Hunan Province, China (Grant No. 2021RC2023, KH), the China Postdoctoral Science Foundation (Grant No. 2021M703638, KH), and the National Natural Science Foundation of China (Grant No. 82201557, KH). |
| 19 | **Data and data sharing** | Provide the data used to perform all analyses or report where and how the data can be accessed, and reference these sources in the article. Provide the statistical code needed to reproduce the results in the article, or report whether the code is publicly accessible and if so, where | 19 | The datasets used and analyzed during the current study are available from the corresponding author on reasonable request. |
| 20 | **Conflicts of Interest** | All authors should declare all potential conflicts of interest | 19 | The authors have no conflict of interest to report. |

This checklist is copyrighted by the Equator Network under the Creative Commons Attribution 3.0 Unported (CC BY 3.0) license.

1. Skrivankova VW, Richmond RC, Woolf BAR, Yarmolinsky J, Davies NM, Swanson SA, et al. Strengthening the Reporting of Observational Studies in Epidemiology using Mendelian Randomization (STROBE-MR) Statement. JAMA. 2021;under review.

2. Skrivankova VW, Richmond RC, Woolf BAR, Davies NM, Swanson SA, VanderWeele TJ, et al. Strengthening the Reporting of Observational Studies in Epidemiology using Mendelian Randomisation (STROBE-MR): Explanation and Elaboration. BMJ. 2021;375:n2233.
