# Supplementary material for: No genetic causal association between dental caries and Alzheimer’s disease: a bidirectional two-sample Mendelian randomization analysis
Source: PeerJ. 2023 Aug 23;11:e15936. doi: 10.7717/peerj.15936 (PMC10460150; doi:10.7717/peerj.15936)
Supplement: Supplemental Information 2 [file peerj-11-15936-s002.docx]

Supplementary files 1: The IDs of GWASs and R codes used in our study

In the two sample MR analysis of effect of dental caries on AD, we selected genetic variants from the most recent meta-analysis by Shungin (1) as exposure. As shown in Supplementary Table 1, we used the data modified from Shungin as the input of exposure files (dental caries) in our analysis, and AD GWAS from as the public the MRC IEU OpenGWAS project. In the two sample MR analysis of effect of dental caries on AD by using GWAS from open-access data provided by the MRC IEU OpenGWAS project. Listed as follows,

| Exposure GWAS ID | Outcome GWAS ID |
| --- | --- |
| Dental caries (local files) | AD (finn-b-G6_ALZHEIMER) |
|  | Early onset AD (finn-b-AD_EO) |
|  | Late onset AD (finn-b-AD_LO) |
|  | AD (ieu-b-2) |
| AD (ieu-b-2) | Dental caries (ukb-b-4770) |

We used the R packages ‘*TwoSampleMR’* *(version 0.5.6)* in our bidirectional two-sample MR analysis, R codes were available online: <https://mrcieu.github.io/TwoSampleMR/index.html>

Reference

1. Shungin D, Haworth S, Divaris K, Agler CS, Kamatani Y, Keun Lee M, et al. Genome-wide analysis of dental caries and periodontitis combining clinical and self-reported data. Nat Commun. 2019;10(1):2773.
